# Physicochemical, Antimicrobial and Antioxidant Properties of Chitosan Films Incorporated with Carvacrol

**DOI:** 10.3390/molecules181113735

**Published:** 2013-11-07

**Authors:** Marco A. López-Mata, Saul Ruiz-Cruz, Norma P. Silva-Beltrán, José de Jesús Ornelas-Paz, Paul B. Zamudio-Flores, Silvia E. Burruel-Ibarra

**Affiliations:** 1 Departamento de Biotecnología y Ciencias Alimentarias, Instituto Tecnológico de Sonora, 5 de febrero 818 sur, Cd. Obregón, Sonora 85000, Mexico; 2 Centro de Investigación en Alimentación y Desarrollo A.C., Av. Río Conchos S/N Parque Industrial, Cuauhtémoc, Chihuahua 31570, Mexico; 3 Departamento de Investigación en Polímeros y Materiales, Universidad de Sonora, Unidad Centro, Hermosillo, Sonora 83000, Mexico

**Keywords:** carvacrol, antibacterial, antioxidant, water vapor permeability, chitosan film

## Abstract

Chitosan films (CF) with carvacrol (CAR) [0.5%, 1.0% and 1.5% v/v] were prepared by the emulsion method. The retained CAR, water solubility, water vapor permeability (WVP), optical, mechanical properties, antibacterial and antioxidant capacity of films were analyzed. The results indicate that the retention of CAR in the CF was ≈50%. The incorporation of CAR to CF decreased the water solubility, the WVP, the yellowing and transparency and the tensile strength, but increased the stiffness. Microcapsules with diameters of 2 to 7 µm were found on the surface CF-CAR. The CF-CAR with highest CAR concentrations showed antibacterial activity against *S. typhimurium* and *E. coli* O157:H7. The CF-CAR had higher antioxidant capacity and an increased protective effect against oxidation of erythrocytes in different grades. These results suggest potential applications of CF-CAR as active packaging to preserve food products.

## 1. Introduction

Currently, consumers demand foods with low levels of synthetic chemical preservatives or even free of them [1]. This phenomenon has increased the search for natural compounds with bioactive properties to keep the quality of different fruits and vegetables in storage. Some alternatives have pointed to the use of matrixes based on carbohydrates, lipids and proteins with film-forming properties [2].

Chitosan is a carbohydrate composed of β-(1-4)-d-glucosamine and β-(1-4)-N-acetyl-d-glucosamine, obtained by alkaline deacetylation of chitin. This viscous polymer is biodegradable, nontoxic, antigenic, antimicrobial and biocompatible [3]. These characteristics have made it ideal for the development of various applications in medicine, agriculture and the food industry [4]. In the latter, chitosan has been used as a natural preservative, due to its film forming properties. Recent applications have shown that chitosan films (CF) can create a semipermeable barrier capable of reducing respiration, microbial growth retardation, maintains firmness and color in fruits and vegetables [5]. In addition, its use has been classified as safe for applications as a preservative in food products [6]. However, a major disadvantage of the CF is its limited activity as moisture barrier because of its hydrophilic nature.

The chitosan polymer matrix has also been used for the incorporation of various bioactive compounds such as vitamins, lipids, minerals, drugs, proteins, dyes, essential oils and phenolic compounds [7]. Carvacrol (CAR) is a phenolic compound found primarily in oils of oregano, thyme, and marjoram, and recognized as a safe additive [8]. This phenolic compound possesses antimicrobial properties, antioxidants, and a particular aroma which makes an attractive ingredient for certain types of foods.

Some of the direct applications of CAR in the food industry are in baked goods, soft drinks and chewing gum [9]. However, direct application to foods, as a preservative, may be limited, since it can be lost during storage due to its high volatility and reactivity with various food components [10]. Although the polymer matrix of chitosan has demonstrated biocompatibility with various compounds, the incorporation of CAR could induce beneficial or adverse effects on the physical-chemical characteristics of the film. Therefore, the purpose of this study was to develop and characterize CF with incorporation of CAR by the emulsion method. The concentration of retained CAR, water vapor permeability (WVP), solubility, and optical properties (color, transparency and transmittance) were determined in the films. The microstructural characteristics of the surface of the films, mechanical properties, antibacterial activity, antioxidant capacity, and protective function of the erythrocyte were also studied.

## 2. Results and Discussion

### 2.1. Microstructural Characterization

**Figure 1:**
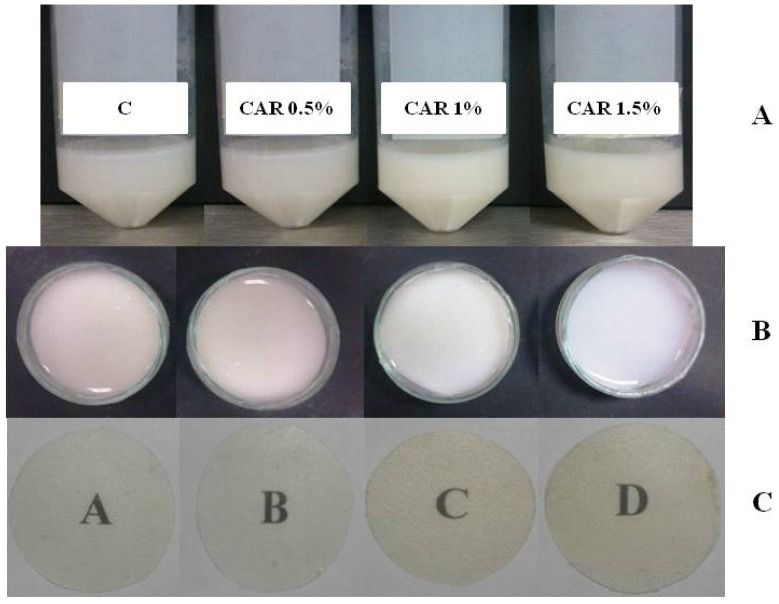
Photographs of the processes preparation of the film: (**A**) emulsions; (**B**) film forming solution in petri dishes; (**C**) film dried.

Initially, the appearance and general physical characteristics of the emulsions and dry film (homogeneity and dispersity of CAR) were visually observed. Homogeneous emulsions were observed as there was no evidence of phase separation due to creaming after 24 h at room temperature. Also, was observed the dispersity of carvacrol in the emulsion system and in the final form of dry film (Figure 1). Similar results were reported by Klikerson and Namatsila [11] for tuna oil-in-water emulsions with chitosan below 2%. Likewise, these authors reported that emulsions with chitosan concentrations above 2% are not homogeneous and that had a layer separation into an opaque cream layer on top and a highly turbid emulsion layer at the bottom. In addition, they observed that there might be some aggregation of the droplets in emulsions, and indicated that the droplets were highly flocculated. Nevertheless, the droplet flocculation in emulsions was disrupted by sonication, leading to the production of stable emulsion. What probably happened in our study, since the application of a homogenization at 15,500 rpm could have caused a stable emulsion and an adequate dispersity of carvacrol in the emulsion system.

**Figure 2:**
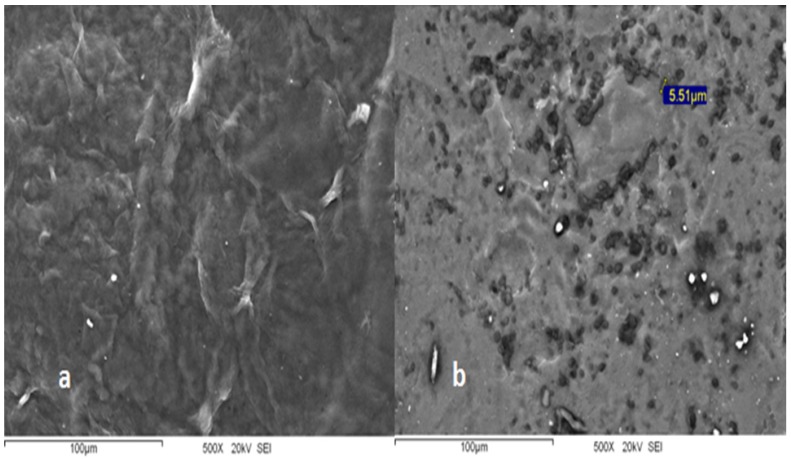
Scanning Electron Microscopy (SEM) pictures of chitosan film (**a**) and chitosan film with 1.5% incorporated carvacrol (**b**) oriented on the dry side.

Figure 2(a) shows the SEM image of a CF (control), the front of evaporation is visually homogeneous and is observed a nonporous surface and irregular shaped folds. Recently, a CF morphology without the presence of pores has been reported [12], although others have documented the presence of uniformly distributed pores [13] on the surface of the film. This phenomenon generally depends on the nature of the chitosan used (degree of acetylation and molecular weight). Furthermore, it is evident that the film surface is modified when CAR is incorporated to chitosan, as shown in Figure 2(b), where the microcapsules are oriented to the dry surface of the film according to the SEM analysis. Moreover, the surface is smoother and spherical microcapsules of approximately 2 to 7 microns in diameter are observed embedded in the film surface. The microcapsule formation may be due to the use of Tween-80. Klikerson and Namatsila [11] reported that the tuna oil was stabilized by Tween-80, because chitosan (positively charged) can adhere to the surface of the droplets covered by Tween-80 (negatively charged) and form droplet aggregates, similar to that observed in the SEM image in our study (Figure 2), whereby, the carvacrol positioning might have affected the hydrophilic/hydrophobic balance of the film. In our view, we show only visual positioning of microcapsules, and a carvacrol physical interaction could not be directly evidenced. This microstructure of CF-CAR could be related to its properties.

### 2.2. Solubility

CF showed the highest percentage of solubility (54.57%, Table 1) compared to CF-CAR films. This high solubility of CF could be attributed to the water binding capacity of the plasticizer (glycerol) and the functional groups of chitosan [14]. Furthermore, it has been previously reported that the use of acetic acid to solubilize chitosan increases its protonation and therefore increases their affinity for water, once the film is formed [12]. The addition of CAR to CF significantly decreases (*p* < 0.05) the solubility of the film between 15% and 23% in a concentration range of 1.5% to 0.5% CAR, respectively. A possible explanation for the decrease of solubility of the films with CAR is that the molecular structure of the chitosan matrix may be diminished by the formation of microcapsules. The lowest water solubility was observed for the film with low concentration of CAR (CF-CAR 0.5%) compared with 1.0% and 1.5%. Similar results were reported by Abdollahi *et al.* [15] in CF incorporated with montmorillonite nanoclay and rosemary essential oil. They observed that the increase in concentration of essential oil of rosemary into film, not necessarily decreases the solubility of the films. Moreover, Rubilar *et al*. [16] reported that chitosan films’ solubility was not affected by the incorporation of carvacrol (0–90 ppm). In order to know the correlation between the solubility and the CAR concentration retention, Pearson correlation analysis was performed. These results show a positive correlation (r = 0.96) between retention of CAR in the films and solubility, which tells us that the greater the retention of carvacrol, the lower the solubility.

**Table 1:** Solubility, WVP and mechanical properties of CF and CF-CAR.

Films	Thickness (mm)	Solubility (%)	WVP (g-mm/m^2^-h-kPa)	TS (MPa)	% E	EM (MPa)
CF	0.116 ± 0.002 ^a^	54.57 ± 0.25 ^a^	1.44 ± 0.04 ^a^	10.169 ± 0.78 ^a^	8.447 ± 0.19 ^a^	3.958 ± 0.96 ^a^
CF-CAR 0.5%	0.111 ± 0.006 ^a^	31.25 ± 0.69 ^b^	1.09 ± 0.04 ^b^	7.596 ± 1.02 ^b^	5.474 ± 0.45 ^b^	2.886 ± 0.41 ^b^
CF-CAR 1.0%	0.108 ± 0.002 ^a^	37.63 ± 0.20 ^c^	1.08 ± 0.02 ^b^	6.384 ± 0.36 ^c^	4.563 ± 0.44 ^c^	2.638 ± 0.46 ^b^
CF-CAR 1.5%	0.112 ± 0.003 ^a^	39.49 ± 0.82 ^c^	1.13 ± 0.01 ^b^	7.821 ± 0.53 ^b^	5.820 ± 0.36 ^b^	3.012 ± 0.52 ^a^

^a–c^ Different letters indicate statistically significant differences between groups as determined by the Duncan test (*p* < 0.05).

### 2.3. Water Vapor Permeability (WVP)

Table 1 shows the WVP of the CF-CAR. The results indicate that incorporation of CAR into CF significantly decreases the WVP compared to the control (*p* < 0.05), and no difference was found when the concentration of CAR increased in the film. This pattern could be related to the presence of microspheres on the surface of the film that produce a more compact network, decreasing the WVP. The WVP results agree with the lower solubility value in the CF-CAR. The decrease in WVP of CF-CAR could be due to modification of the hydrophobic portion of the film, as a result of the ratio between chitosan and CAR [17]. Similar results were reported by Rubilar *et al*. [16], on chitosan films with carvacrol (60–90 ppm) and grape seed extract (160–400 ppm). In contrast, Du *et al*. [18] found that the addition of CAR (0.5%, 1.0% and 1.5%) to tomato puree films increased WVP compared to the control. The drawback is that they added the CAR to high temperature (100 and 132 °C) during the preparation of the films, and this may have increased the evaporation or destruction of CAR, which may have affected in the WVP.

In the same sense, it was reported a reduction up to 50% for CF incorporated with bergamot oil [19]. Furthermore, it has been found that films of casein and lipid material (beeswax) may reduce WVP up to 70%. One limitation that should be considered when adding hydrophobic compounds at high concentrations is that it could contribute to hydro-repulsion, which would limit its application in foods with high moisture content. It has also been documented that it may impact on the sensory, mechanical and optical properties of the films [7].

### 2.4. Mechanical Properties

The effect of the incorporation of CAR in different concentrations on the mechanical properties of CF is presented in Table 1. CF (control) presented values of 10 ± 0.78 MPa, 8.447% ± 0.19% and 3.958 ± 0.96 MPa for TS, %E and MB, respectively. These values are lower than those reported by Rubilar *et al*. [16], (TS = 48 MPa and EB = 28%). This could be due to factors such as the source of chitosan, great dispersion of the respective degrees of deacetylation, molecular weights, use of a plasticizer, film preparation and storage [20]. The incorporation of CAR at all concentrations showed a significant decrease (*p* < 0.05) in TS, %E and EM compared with control. However, the carvacrol incorporation in 1% concentration showed a significant decrease on the TS and %E of the chitosan films. This is probably due to the major percentage of retention of carvacrol on this film. Since it has been proposed that the addition of hydrophobic agents to the film composition could be induce the development of a structure with less mobility and therefore less flexibility and resistance to fracture [5,21]. Similar results were reported by Rubilar *et al*. [16] and Martins *et al*. [20] on chitosan films with carvacrol and α-tocopherol, respectively. Regarding EM, CF-CAR is stiffer than the control film. Similar pattern was observed by Rubilar *et al*. they found that incorporation of CAR and grape seed extract into CF decreased TS and %E values [16].

### 2.5. Optical Properties

The color and whiteness index (WI) measurements are shown in Table 2. In general, the films showed a tendency to yellow (b*). This trend decreased significantly for all films incorporated with CAR compared to control (*p* < 0.05). The presence of a yellow color may be considered as a natural characteristic in the CF, since this color has been associated with the presence of repeat units of β-(1-4)-2 amino-2-deoxy-d-glucopyranose [13]. The values of L* (luminance) significantly decreased in the films with CAR compared to control (*p* < 0.05). In general, the values of L* are low compared to those reported in CF added with particles (range values of 66–93) [12]. Currently, there is controversy concerning the color values obtained from CF because the chitosans used in different studies often come from different sources, and the processes for obtaining them were different [22]. On the other hand, incorporation of CAR into the CF did not affect the a* (red-green) value, but reduced the b* (yellow-blue) value, still retaining the yellow coloration. A similar pattern was observed by Casariego *et al*. [12] in clay-CF. In regard to the control film, the WI was greater than those containing CAR, this indicates that the whiteness of the film significantly decreased (*p* < 0.05) when CAR was added. Although WI decreased by adding CAR, generally the films still have a high whiteness compared to previously reported values of 50 for WI in CF [13]. Previously has been reported that surfactants agents such as Tween-20, are not able to modify the transparency of the chitosan films, but could be influence an increase in yellow color, which is associated with the yellow color of Tween [23]. In our study, the control (CF) show the higher yellow color compared with CF-CAR films, this indicates that the Tween-80 was not probably a significant factor.

**Table 2:** Color and WI parameters of CF and CF-CAR films.

Films	L*	a*	b*	WI
CF	59.47 ± 0.14 ^a^	−1.30 ± 0.01 ^a^	14.69 ± 0.19 ^a^	70.44 ± 0.23 ^a^
CF-CAR 0.5%	52.59 ± 1.27 ^b^	−0.45 ± 0.04 ^a^	10.09 ± 0.19 ^b^	68.44 ± 0.23 ^b^
CF-CAR 1.0%	54.05 ± 0.18 ^c^	−1.42 ± 0.01 ^b^	12.91 ± 0.01 ^c^	69.52 ± 0.40 ^c^
CF-CAR 1.5%	53.07 ± 0.10 ^b^	−1.37 ± 0.13 ^b^	10.02 ± 0.70 ^b^	69.19 ± 0.02 ^c^

^a–c^ Different letters indicate statistically significant differences between groups as determined by the Duncan test (*p* < 0.05).

The transparency of a film is a desirable property because the consumer needs to clearly see the appearance of the product [24]. Table 3 shows the behavior of the transparency of CF and CF-CAR, where observed that the addition of CAR to the CF at concentrations of 1.0% and 1.5% had a significant reduction in the transparency, between 1.6%–2.0%, respectively. In general based on the L* and transparency values, the films can be considered as opaque, and this is due to the nature of the chitosan more than the addition of CAR.

**Table 3:** Light transmittance (%) and transparency values of CF and CF-CAR films.

Films	Waveslength (nm)								
	200	280	350	400	500	600	700	800	Transparency Values
CF	0.0011	0.8125	1.41	14.65	27.80	31.68	33.50	34.36	2.49 ± 0.001 ^a^
CF-CAR 0.5%	0.0007	0.0019	5.40	17.30	30.81	35.46	38.40	40.40	2.54 ± 0.001 ^b^
CF-CAR 1%	0.0006	0.0018	1.70	10.23	24.19	28.08	29.90	31.00	2.45 ± 0.002 ^c^
CF-CAR 1.5%	0.0007	0.0019	2.90	13.27	25.48	28.07	29.31	29.94	2.44 ± 0.001 ^c^

^a–c^ Different letters indicate statistically significant differences between groups as determined by the Duncan test (*p* < 0.05).

The light transmission of the film in a wavelength range from 200 to 800 nm was 1.41%–40.4% in the range of visible wavelength (350–800 nm) (Table 3). Transmission of CF in the UV range (200–280 nm) decreased with addition of CAR at the three concentrations used. This indicates that the CF-CAR can block the UV light transmission toward the product with this film. It was reported that some films made of animal gelatin can block the UV light more effectively than the films of synthetic origin [25].

### 2.6. Quantification of CAR Incorporated in CF

**Figure 3:**
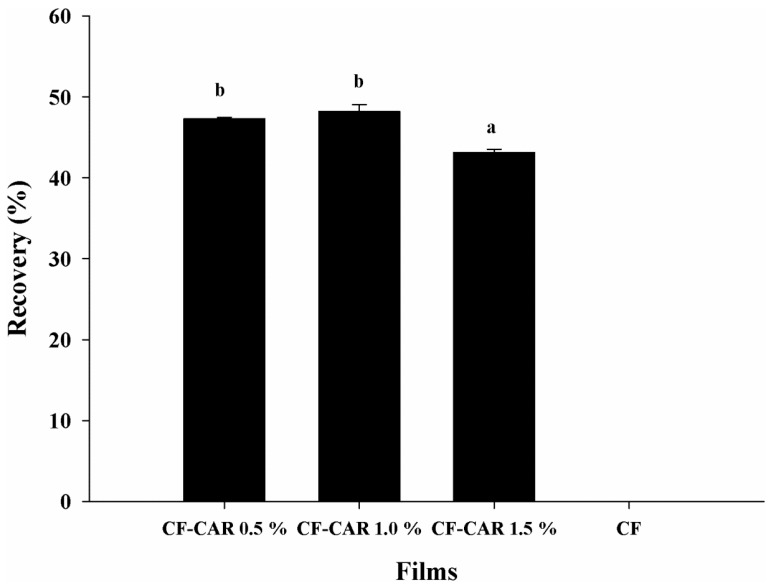
Percentage of CAR retention in the chitosan films. The data are the mean values of at least three determinations. The mean values represented by the bars for each type of films extract that are indicated with a different letter are significantly different (*p* ≤ 0.05).

CAR decomposition and volatilization can easily occur during heating processes, application of pressure or light, or the presence of oxygen. These are all factors commonly used during food processing. In this sense, the addition of CAR to CF emerges as an important alternative for retaining its functional properties. In this study, the 0.5% to 1.0% CF-CAR preparations showed a respectively higher retention of CAR [47.2% (0.0461 ± 0.0009 g/g TWF) and 48.2% (0.101 ± 0.0017 g/g TWF)], compared to the 1.5% CF-CAR [43.1% (0.136 ± 0.0010 g/g TWF)] (*p* < 0.05) (Figure 3). The loss of CAR could be influenced by diverse physical processes that were used in this study (homogenization, centrifugation and temperature), which resulted in the release of the bioactive compound, where the stability of the emulsion plays an important role [26]. Although we did not evaluate the effects of changes during the drying phase and its conditions, there has recently been evidence during the initial process in the development of film-forming solution with added carvacrol, during processing and drying (solvent release) can result in carvacrol losses from chitosan films between 50%–99% [27]. This author also reports that dry films can release carvacrol between 0.04% to 10.75%, but this variation will depend on the structure of the film, its composition and the conditions under which the film is processed. Regarding the limitation of the previous study, we did not use surfactants (Tween-80) to stabilize the emulsion. Furthermore, Higueras *et al*. [28] have reported that the relative humidity (0%, 53%, 75% and 90% RH) and glycerol used at two different concentrations (20 to 35 g of dry matter glycerol/100 g) had low carvacrol absorption (0.08 to 0.96 g of dry matter carvacrol/100 g). Unlike films prepared from chitosan-hydroxypropyl-β-cyclodextrin (1:1 w/w) glycerol at 53% and 75% RH showed a high uptake of carvacrol (133–216 g/100 g dry matter, respectively). The above differs methodologically from our study as these authors studied the submersion of the film formed on a solution of carvacrol (up to 3 months), waiting for their absorption, in contrast to our study, where we used a film forming solution of chitosan with glycerol as a plasticizer, then added the Tween-80 carvacrol, which could have influenced the differences in carvacrol recovery. The CAR content in the CF reported in our study may be considered high compared to the study of Keawchaoon and Yoksan [29], who used a method of emulsifying and ionic gelation of chitosan with pentasodium tripolyphosphate to produce chitosan nanoparticles loaded with CAR. They reported values between 3% and 21% which are considered as a low CAR loading capacity. On the other hand, Liolios *et al*. [30] used the liposomal encapsulation method, achieving only a 4.16% encapsulation of CAR. Therefore, the microencapsulation of CAR may result in a greater stability of the molecule.

### 2.7. Antibacterial Activity

The antibacterial activity of CF-CAR against two major food contaminant pathogenic bacteria (*E. coli* O157:H7 and *Salmonella*) was evaluated. *Salmonella* showed sensitivity against CF-CAR at 1.0% and 1.5% (Table 4). In contrast, *E. coli* O157:H7 was sensitive to CF-CAR with 1.5%. Although 0.5% CF-CAR tested against *Salmonella* and 0.5% and 1% for *E. coli* O157:H7 had no sensitivity based on the used classification, there was an inhibitory effect under the film. It has been previously reported that CAR can inhibit the development of *Salmonella* (0.5 mmol/L) and *E. coli* (1 mmol/L) [31,32]. The antibacterial mechanism of CAR has been studied already and it is known that it is due to the interaction with lipophilic components of the bacterial membrane. This can cause changes in the permeability of H^+^ and K^+^, which can damage the essential functions and cause cell death [33]. Olasupo *et al*. [34] reported that the minimum concentration of CAR needed to inhibit the growth of *E. coli* (1.5 mmol/L) is greater than the concentration needed to inhibit *Salmonella* (1 mmol/L). This could explain why we saw no inhibitory effect of CF-CAR 1.0% against *E. coli* O157:H7 while *Salmonella* was sensitive. Another explanation of the absence of inhibition in CF-CAR with the lowest CAR concentrations could be due to the fact that microcapsules (observed in SEM) are not releasing the CAR content and the effect shown at high concentrations may be due to the presence of residual CAR in the CF and not to the encapsulated one.

**Table 4:** Antibacterial activity expressed as the inhibition zone diameter (mm) of chitosan films against *S.* *typhimurium* and *E. coli* O157:H7.

Films	Inhibition Zone * (mm)	
	*S.* *typhimurium*	*E. coli* O157:H7
CF	<8.0	<8.0
CF-CAR 0.5% (0.7592 g df)	<8.0	<8.0
CF-CAR 1.0% (0.8206 g df)	9.8 ± 0.14	<8.0
CF-CAR 1.5% (0.8700 g df)	11.7 ± 0.10	9.8 ± 0.16

* Mean ± standard error; df = dry film.

### 2.8. Antioxidant Capacity

#### 2.8.1. Radical Scavenging Capacity Using the DPPH Method

**Figure 4:**
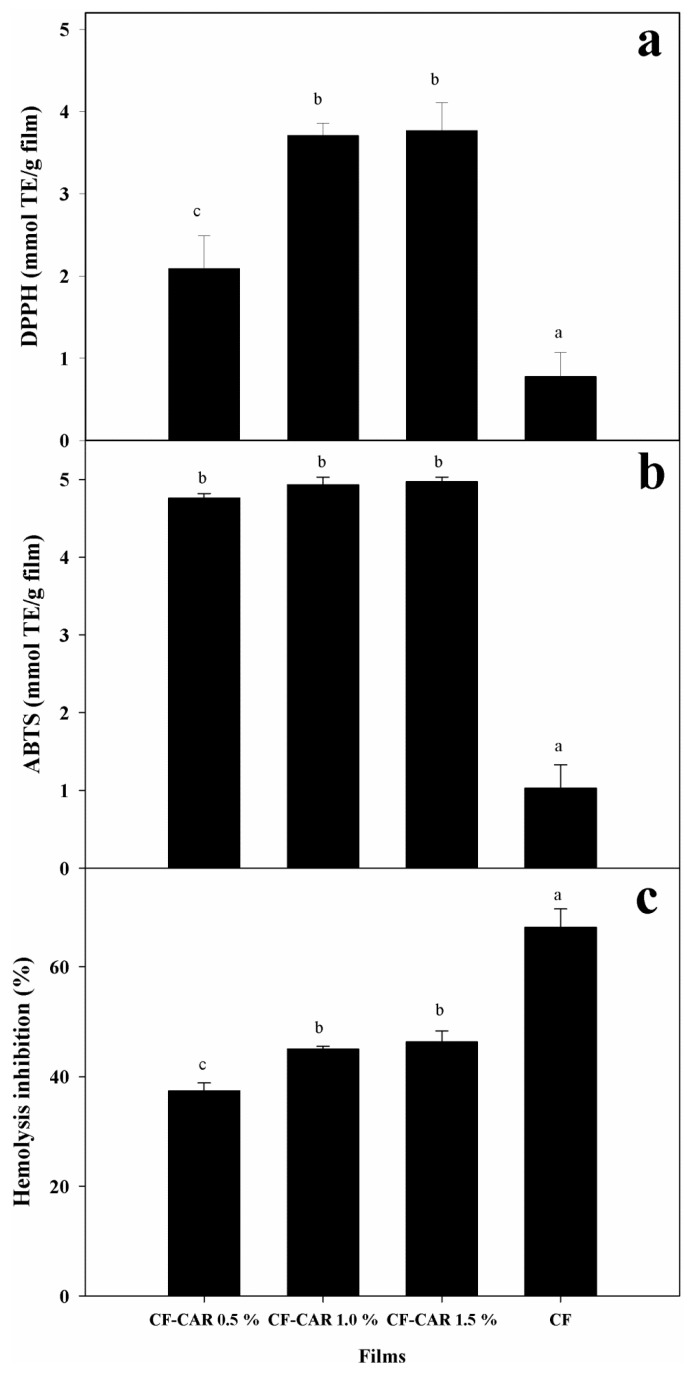
Antioxidant capacity of films extract determined by DPPH (**a**), ABTS radicals (**b**) and percentage of hemolysis inhibition (**c**). The data are the mean values of at least three determinations.The mean values represented by the bars for each type of films extract that are indicated with a different letter are significantly different (*p* ≤ 0.05).

The importance of the incorporation of antioxidants, such as CAR, into CF has the advantage that it can act as a stabilizer of the food components. The oxidation of lipids is one of the major causes of food spoilage and takes place on the food surface [35]. Figure 4(a) shows the antioxidant capacity of the CF-CAR extracts compared to the CF control. The CF-CAR extract showed more activity than the control sample (*p* < 0.05). These CF-CAR extracts have antioxidant capacity, which is intensified by incorporating CAR. Likewise, it was observed that such capacity is increased at a rate of (CF-CAR/CF) of 4.8 (CF-CAR 1.5%), 4.7 (CF-CAR 1%) and 2.6 (CF-CAR 0.5%), respectively.

#### 2.8.2. Trolox Equivalent Antioxidant Capacity (TEAC)

Figure 4(b) shows the antioxidant capacity of the CF-CAR extracts to capture the radical ABTS. We did not find differences in antioxidant activity in any of the tested CF-CAR extracts. The antioxidant capacity was 4.82 (CF-CAR 1.5%), 4.78 (CF-CAR 1%), 4.62 (CF-CAR 0.5%) and 1.08 (CF) control. The antioxidant capacity of chitosan has been previously documented. The free radical capturing activity of chitosan has been attributed to the presence of a protonated nitrogen on carbon number 2, which has the ability to simultaneously bind several free radicals [36]. Some authors have shown that the molecular weight of the chitosan is also an important factor in its antioxidant capacity [37,38]. Furthermore, the antioxidant capacity of CAR depends on the steric and electronic effect of its ring, besides the presence of the hydroxyl group, which is capable of donating hydrogen atoms [39]. Further investigations have shown that the incorporation of antioxidant in the formulation of the film may function as a barrier to oxygen, which results in a better preservation of the quality of the food product [40].

#### 2.8.3. Evaluation of the Protective Effect on Human Erythrocytes

Figure 4(c) shows the effect of CF and CF-CAR film extracts on the inhibition of hemolysis. All the film extracts showed a protective effect against erythrocyte hemolysis. The highest percentage of inhibition of hemolysis corresponded to CF control extracts (67.2%), which was significantly higher than even the CF-CAR extracts at 0.5% (37.4%), 1% (45%) and 1.5% (46.3%), respectively. It has been observed that AAPH is an initiator of peroxyl/alkoxy radicals at physiological temperature in the presence of oxygen. These radicals have been identified as the principal particles responsible for the biological damage in cells [41,42]. Some authors have reported that in acidic solution, the amino groups in chitosan are protonated, which induces an electrostatic interaction with the negatively charged membrane surface of the erythrocyte, producing cellular aggregation, but this aggregation generally causes no serious damage to the erythrocyte cell membrane [43,44]. Our results suggest that aggregation of erythrocytes can form a protective polymeric network against peroxyl radicals. This can explain the higher inhibition of hemolysis by the control film than CF-CAR at all concentrations. Results from chemical methods that were used to measure the antioxidant capacity (TEAC and DPPH) of our films differ in the protective effect of chitosan against free radicals. One possible explanation may be that CAR acts more directly with the radicals generated by AAPH and it does not form a protective aggregation like chitosan does.

## 3. Experimental

### 3.1. Materials

CAR (98% purity), 2'2-azino-bis-(3-ethylbenzothiazoline-6-sulfonic acid) (ABTS), 2,2-diphenyl-1-picrylhydrazyl (DPPH) radical, 6-hydroxy-2,5,7,8-tetramethylchromane-2-carboxylic (97% purity), potassium persulfate, 2,2'-azobis (2-methylpropionamidine) dihydrochloride (AAPH; 97% purity) and sodium bromide (NaBr) were purchased from Sigma-Aldrich (St. Louis, MO, USA). Water, methanol, ethanol and acetonitrile (HPLC grade) were purchased from JT Baker (St. Louis, MO, USA). The chitosan was prepared from shrimp (*Penaeus vannamei*) chitin. All other reagents used in this study were of analytical grade.

### 3.2. Preparation of Chitosan

The chitosan was obtained by thermo-alkaline deacetylation of chitin. Chitin (1 g) was homogenized with 50% w/v NaOH (15 mL) at 95 °C for 2 h [45]. The degree of acetylation of chitosan used in this study was 34%, with an average molecular weight of 128 kDa as previously described [46].

### 3.3. Preparation of CF with Incorporation of CAR

The preparation of CF with incorporation of CAR was performed as follows: chitosan (0.4 g) was dissolved in 1% v/v acetic acid (20 mL) and glycerol (500 µL/g of chitosan) and homogenized for 4 min at 15,500 rpm in an Ika Ultra-turrax T 18 basic (Staufen, Germany). Thereafter, an emulsion was prepared with chitosan coating solution, (1% v/v) Tween 80, (50% v/v) ethanol-water and CAR. CAR was incorporated at the following concentrations: 0 (control), 0.5 (0.097 g), 1.0 (0.211 g), and 1.5% (0.316 g) (v/v). The emulsions were homogenized as previously described and kept at 5 °C overnight. The emulsions were poured into glass Petri dishes and oven-dried (37 °C × 24 h at 0% HR). Dried films were peeled off and stored in a desiccators containing anhydrous calcium sulfate (0% HR at 25 °C) before use for further testing.

### 3.4. Thickness

The film’s thickness was measured with a micrometer (Mitutoyo, Tokyo, Japan) with accuracy if ±0.001 mm. Ten thickness measurements were taken on each testing sample in different points and the mean values were used for transparency and water vapor permeability calculations.

### 3.5. Scanning Electron Microscopy

Surface morphology of the samples was examined using a Scanning Electron Microscope (SEM, JEOL JSM-5410LV) (JEOL-LTD, Tokyo, Japan), equipped with an INCA system and an energy dispersive X-ray detector (Oxford Instruments, Buckinghamshire, UK), and operated at a voltage of 20 kV. For SEM analysis, the specimens were cut to an appropriate size to be mounted on a sample holder, which has se cylindrical form, is made of copper and 1 cm in diameter. The samples were glued to the sample holder using carbon double-sided stick tape and gold coated to provide conduction and prevent charging under electron bombardment [47]. Observation of the sample was performed under high vacuum using the secondary electron detector.

### 3.6. Water Solubility

The film solubility in water was determined according to the method reported by Casariego *et al.* [12]. Four rectangular pieces (7.5 mm × 15 mm) were cut from each film and dried under vacuum in an oven at 105 °C and 35 kPa for 24 h (to a constant weight) to determine the initial dry weight and then the films were immersed in 50 mL of water at 20 °C with occasional agitation. After 24 h of immersion, the specimens of films were recovered by gently rising with distilled water and then dried to constant weight (105 °C). The measurement of films solubility was determined as follow:

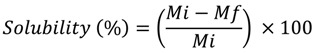
(1)
where *Mi* and *Mf* are the initial and final mass of the sample.

### 3.7. Water Vapor Permeability (WVP)

WVP of the films was determined gravimetrically based on the ASTM E96-92 method [48]. The tested films were sealed on top of a permeation cell containing distilled water (100% RH; 3.168 kPa vapor pressure at 25 °C inside) and placed in a desiccator (0% RH; 0 kPa vapor pressure-outside) with anhydrous calcium sulfate. The water transferred through the film and absorbed by the desiccant was determined from weight loss of the permeation cell (measured every 2 h during 10 h). Weight loss over time was plotted to obtain the slope (R^2^ = 0.995). The slope was used to calculate water vapor transmission rate (WVTR) using the following equation:

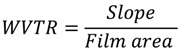

where *Slope* = weight loss *vs*. time and *Film area* = cup test mouth area.

The WVP was calculated as follows:

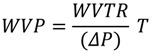
(2)
where *WVTR* is the measured water vapor transmission rate, *T* is the mean film thickness and *ΔP* is the partial water vapor pressure difference across the two sides of film.

### 3.8. Mechanical Properties

Standard method ASTM-D882-95 [49] was used to measure tensile strength (TS), % elongation (%E) and elasticity modulus (EM). Films were cut into rectangles (6 × 1 cm) and conditioned at 52% relative humidity (RH) for 24 h before testing using NaBr. After, the films were mounted and clamped with grip of Texturometer TAXT-Plus (Stable Micro Systems, Surrey, UK). The results were analyzed with Exponent Lite Software (Version 4.0). The conditions of the test were with 30 kg load, initial gauge length of 4 cm and stretched using a crosshead speed of 20 mm min^−1^. TS was expressed in MPa and calculated by dividing the maximum load by initial cross-sectional area of film. %E was calculated as the ratio of increased length to initial length of film and expressed as a percentage. EM was evaluated for the slope of stress-strain lineal behavior. All tests were replicated five times for each type of film.

### 3.9. Optical Properties

Color, transparency and transmittance of films were determined with a Cintra 10e (GBC Scientific Equipment, Victoria, Australia) spectrophotometer. Color parameters *CIE* L* (lightness), a* (red-green) and b* (yellow-blue) were calculated using illuminant D65 and 10° observer angle (white standard plate was used for calibration). Film samples were measured and the results were expressed as average of six samples. The whiteness index (*WI*) was determined as follows:

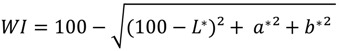
(3)

Film transparency was determined using the method of Han and Floros [50]. Films were cut into rectangular shapes (9 × 45 mm) and placed on the internal side of the spectrophotometer cell at 600 nm. Transparency of films was measured by three replicates of each film. The percentage transparency was calculated as follows:

*Transparency value* = log (*T*_600_/*T*) (4)

where *T*_600_ is the transparency at 600 nm and *T* is film thickness (mm).

UV and visible light transmission barrier properties were determined by measuring their light absorption at wavelengths from 200–800 nm according to the method described by Pereda *et al*. [13].

### 3.10. Measurement of CAR Retained in Films by HPLC

Film extracts were prepared according to Du *et al*. [18]. Film (50 mg) was homogenized with 50% (v/v) ethanol (5 mL) during 5 min at 11,000 rpm. The extract was filtered through a 0.45 µm nylon membrane and volume sample of 10 µL were injected into a Waters 2795 analytical HPLC system consisting of a quaternary pump and UV-visible detector (Waters 717 plus). An Xterra^®^ C_18_ column (4.6 × 50 mm) packed with 5 μm particle. The following conditions were used: the eluent consisted in acetonitrile and water 55:45 v/v with a flow rate of 0.75 mL/min. The absorbance was monitored at 277 nm. Analyses were performed in triplicate with a variation <5%. A carvacrol curve was determined (R^2^ = 0.998) and the results were expressed as g CAR/g of total weight of the film and the percentage of retention in the films and were calculated using the following equation:

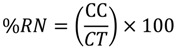
(5)
where *%RN* = retention percentage, CC = calculated concentration and *CT* = theoretical concentration.

### 3.11. Antibacterial Activity

*Escherichia coli* O157:H7 (ATCC 43890) and *Salmonella* *typhimurium* (ATCC 14028) were utilized. The agar diffusion method was used to determine the bacterial sensitivity when exposed to the films. An inhibition zone assay was performed adding 100 µL of inoculate containing 10^5^ cfu/mL of each tested strain and streaked out over the surface of Muller-Hinton agar plates (Difco, Edo. México, México). Different films were cut into 6 mm diameter discs and then placed on inoculated agar (incubated at 37 °C for 24–48 h) as described by Coma *et al*. [51]. Sensitivity was classified according to diameter of the halo as: not sensitive (<8 mm), sensitive (9–14 mm), very sensitive (15–19 mm) and ultrasensitive (>20 mm) [52]. One control of CF without CAR was used. Inhibition zone assays were performed in triplicate.

### 3.12. Antioxidant Capacity

#### 3.12.1. Radical Scavenging Capacity Using DPPH Method

Films (50 mg) were homogenized with methanol (80%, 5 mL) during 5 min at 11,000 rpm and then centrifuged at 1,200 ×*g* during 10 min at 5 °C and filtered (Whatman #1 paper). Methanolic film extracts were used to perform DPPH, ABTS and hemolysis assays. Free radical-scavenging capacity was measured according to the method of Moein and Moein [53], DPPH radical was prepared at 0.025 g/L with methanol and solution was adjusted to an absorbance of 0.7 ± 0.02 at 490 nm. Trolox (6-hydroxy-2,5,7,8-tetramethylchromane-2-carboxylic acid) was used as a standard and 80% methanol as a blank. Film extract (5 µL) was added to the DPPH (245 µL) and incubated at 25 °C for 30 min in darkness. The absorbance was then read in an iMark micro-plate reader (Bio-Rad, Tokyo, Japan). The percentage of inhibition was calculated and results were expressed as Trolox equivalents (mmol TE/g film).

#### 3.12.2. Trolox Equivalent Antioxidant Capacity (TEAC)

The ABTS cation was generated through the interaction of ABTS (19.2 mg) dissolved in water (5 mL) and potassium persulfate (0.139 mM, 88 µL). This mixture was incubated in the dark at room temperature for 16 h. Thereafter, ABTS activated radical (1 mL) was taken and ethanol (88 mL) was added. The solution was adjusted at an absorbance of 0.7 ± 0.02 nm at 734 nm. The TEAC value of film extract was determined according to Re *et al*. [54]. For the assay, ABTS (245 µL) and film extract (5 µL) were mixed and absorbance was monitored at 1 and 10 min in a Bio-Rad iMark micro-plate reader. The percentage of inhibition was transformed to Trolox equivalents (mmol TE/g film).

#### 3.12.3. Evaluation of Protective Effect on Human Erythrocytes

Hemolysis induced by AAPH [2,2'-azobis(2-methylpropionamidine) dihydrochloride] was determined by the method of Lu *et al*. [55] with the following modifications. The erythrocytes were washed threes time with phosphate buffer saline (PBS) pH 7.4, then a suspension of human erythrocyte in (5%) PBS was prepared. Erythrocyte suspension (50 µL), film extract (50 µL) and AAPH (400 mmol) were added into a test tube. A similar reaction mixture was prepared without film extract (complete hemolysis). The mixture was incubated at 37 °C with continuous shaking (30 RPM) in the dark for 3 h. After incubation, the reaction mixture was diluted with PBS (1 mL) and centrifuged (2,000 ×*g* for 5 min). The absorbance of supernatant was measured at 540 nm in a Bio-Rad iMark micro-plate reader. Percentage of hemolysis inhibition (PHI) was calculated as follows:

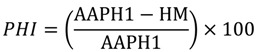
(6)
where AAPH1 = absorbance of complete hemolysis; HM = supernatant of film extract.

### 3.13. Statistical Analysis

The experimental data was subjected to analysis of variance (ANOVA) using the NCSS Statistical Analysis System (Kaysville, UT, USA). Results were expressed as the mean value ± standard deviation (SD). The analysis of variance and significant differences were analyzed by Duncan’s multiple range tests at a 0.95 confidence level.

## 4. Conclusions

A considerable amount of CAR was present (≈50%) in the CF-CAR. This retention of CAR in the CF can significantly decrease the solubility and reduce the loss of water vapor and TS, but increases the stiffness of the film. The films showed a tendency to yellowing, with a slight opacity and less WI than the CF. Furthermore, it was found that CF-CAR can block UV light. Furthermore, the CF-CAR presented antibacterial activity as measured by the agar diffusion method and antioxidant capacity. The CF showed a better ability to protect erythrocytes against oxidative attack. The improvement and conservation of bioactive components in the CF involve a step in the formation of new active packaging.
